# CALCR exacerbates renal cell carcinoma progression via stabilizing CD44

**DOI:** 10.18632/aging.205586

**Published:** 2024-07-09

**Authors:** Haiyang Yan, Zhaohui Xing, Shuai Liu, Peng Gao, Qingli Wang, Guiying Guo

**Affiliations:** 1 Department of Urology, The First Affiliated Hospital of Harbin Medical University, Harbin, Heilongjiang 150001, China; 2 Department of Urology, Heilongjiang Provincial Hospital, Harbin, Heilongjiang 150036, China; 3 Department of Urology, The Third Affiliated Hospital of Qiqihar Medical College, Qiqihaer, Heilongjiang 161099, China

**Keywords:** CALCR, RCC, CD44, ubiquitination, proliferation

## Abstract

The calcitonin receptor (CALCR) is an essential protein for maintaining calcium homeostasis and has been reported to be upregulated in numerous cancers. However, the molecular role of CALCR in renal cell carcinoma (RCC) is not well understood. In this study, we identified the overexpression of CALCR in RCC using human tissue chip by immunohistochemical (IHC) staining, which was associated with a poor prognosis. Functionally, CALCR depletion inhibited RCC cell proliferation and migration, and induced cell apoptosis and cycle arrest. CALCR is also essential for *in vivo* tumor formation. Mechanistically, we demonstrated that CALCR could directly bind to CD44, preventing CD44 protein degradation and thereby upregulating CD44 expression. Moreover, a deficiency in CD44 significantly attenuated the promoting role of CALCR on RCC cell proliferation, migration and anti-apoptosis capacities. Collectively, CALCR exacerbates RCC progression via stabilizing CD44, offering a fundamental basis for considering CALCR as a potential therapeutic target for RCC patients.

## INTRODUCTION

Renal cancer, which is also referred to as renal cell carcinoma (RCC), is a malignant tumor that originates from the renal tubular epithelium [1]. Histologically, renal cancer is categorized into clear cell RCC, papillary RCC, chromophobe RCC and renal oncocytoma [2]. Among these subtypes, clear cell RCC is the most common and aggressive, accounts for approximately 75% of all cases [3]. Despite advancements in imaging techniques leading to improved early detection, it is unfortunate that around 30% of RCC cases are already in advanced stages or have metastasized at the time of diagnosis [1]. Over the past decade, the use of molecular-targeted drugs and the immune checkpoint inhibitors has become vital in the treatment of metastatic RCC [4]. However, these systemic treatments often come with significant side effects and demonstrate varying efficacy, yielding positive outcomes for only a subset of patients [5]. Therefore, it is crucial to uncover the molecular mechanisms underlying RCC pathogenesis and metastasis in order to identify more effective targets for the development of medical therapies.

Calcitonin receptor (CALCR) is a gene that encodes a protein responsible for inhibiting bone reabsorption and enhancing renal calcium excretion [6]. CALCR exhibits widespread expression in a plethora of tissues throughout the life cycle and under different conditions including cell stress, inflammation and in a range of diseases [7]. The CALCR gene has two isoforms, CALCR insert-negative and CALCR insert-positive [8]. The insert positive form is located intracellularly, whereas the insert negative form is found on the plasma membrane [9]. Studies have reported that CALCR plays a crucial role in maintaining quiescence in muscle stem cells [10]. CALCR gene polymorphisms is involved in calcium urolithiasis in Russian population [11] and kidney stone disease in Egyptians [12]. Additionally, CALCR expression in the medial amygdala is associated with social contact of females [13]. Moreover, CALCR is also found to be expressed in a number of cancer cell lines such as breast cancer, bone cancer, prostate cancer, multiple myeloma, leukemia and glioblastoma [14]. However, the molecular role of CALCR in cancers, particularly RCC, remains poorly understood, with limited insight into its specific functions and implications in RCC. Further research is needed to elucidate the molecular mechanisms through which CALCR contributes to the development and progression of RCC. Understanding the role of CALCR in RCC may provide valuable insights for the development of targeted therapeutic approaches for this type of cancer.

Herein, we conducted an investigation of the alterations in CALCR expression in patients diagnosed with RCC and shed light on the biological implications of CALCR in the development of this type of cancer. In particular, we demonstrated the mechanistic role of CALCR in CD44 protein stability. Our results also suggested that JNK and STAT1 signaling were possible signaling pathways that contributed to CALCR-mediated renal carcinoma progression. These discoveries suggest that targeting CALCR could hold promise as a potential therapeutic target for the treatment of RCC.

## MATERIALS AND METHODS

### Tissue microarray and immunohistochemical (IHC) analysis

A human tissue microarray chip containing 79 cases of RCC tissues and 73 cases of normal para-carcinoma tissues was used to identify CALCR protein expression through IHC staining. The tumor characters of each patient and the written informed consents were obtained. This study was approved by the Ethics Committee of The First Affiliated Hospital of Harbin Medical University. For IHC staining, tissue slides were dewaxed and rehydrated using xylene and alcohol. Following this, citrate buffer was added to repair antigens, and 3% H_2_O_2_ was used to block nonspecific binding sites. The primary anti-CALCR (1:50, Cat No. ab230500, Abcam) and secondary HRP goat anti-rabbit IgG (1:400, Cat No. ab97080, Abcam) were then incubated with tissue slides. Finally, slides were stained with diaminobenzene (DAB) and hematoxylin followed by photographing. Two independent pathologists scored the IHC figures according to Haonon et al. [15]. Briefly, the staining score was determined as product of staining intensity and the percentage of positive tumor cells. Staining intensity was divided into four levels: no staining signals (0), light yellow (1), pale brown (2), seal brown (3). The percentages of positive tumor cells were also divided into 4 levels: 0~24% (1), 25~49% (2), 50~74% (3), 75~100% (4). The product of these scores resulted in the immunoreactive score (IRS) ranged from 0 to 12: 0 score (low expression), 1–3 scores (weak expression), 4–7 scores (moderate expression), 8–12 scores (strong expression).

### Cell culture and treatment

The human RCC cell lines 786-O and ACHN were all purchased from Cell Bank of the Chinese Scientific Academy (Shanghai, China). The 786-O cells were cultured in RPMI-1640 medium (Gibco, USA) and the ACHN cells were grown in DMEM-H medium (Gibco, USA). The culture medium for both cell lines was supplemented with 10% fetal bovine serum (FBS) (Gibco, USA) and 1% Penicillin/Streptomycin (100 U/mL). The cells were maintained in a 5% CO_2_ incubator at 37°C.

### RNA interference, overexpression and cell transfection

The knockdown of CALCR and CD44 was completed by constructing respective shRNA sequences. In brief, three shRNA sequences targeting the human CALCR or CD44 gene were designed and synthesized, named shCALCR-1/2/3 or shCD44-1/2/3, according to the manufacturer’s instructions. The shCALCR-1/2/3 or shCD44-1/2/3 plasmids were then generated by inserting their interfering sequences into BR-V108 vector plasmid (Yibeirui, China). After identifying positive clone plasmids, shCALCR and shCD44 plasmids were co-transfected with the pMD2.G (Qiagen, China) and pSPAX2 (Qiagen, China) packaging plasmids into 293T cells to produce shCALCR and shCD44 lentivirus. Similarly, CALCR overexpressing sequence was inserted into LV-007 (Yibeirui, China) vector plasmid and its lentiviral vector was generated by co-transfecting LV-007, pMD2.G and pSPAX2 plasmids into 293T cells. Plasmids carrying scramble sequences and the empty vector were used as negative controls for shCALCR/shCD44 and CALCR, respectively. Targeting sequences for shRNA mentioned above were shown in Supplementary Table 1.

For cell transfection, 786-O and ACHN cells were seeded into 6-well plate at a density of 2 × 10^5^ cells per well for 24 h. The indicated lentiviruses were then added to infect 786-O and ACHN cells using Lipofectamine 2000 (Thermo Fisher Scientific, USA) at a multiplicity of infection (MOI) of 8 and 15, respectively. Transfection efficacy was determined by qPCR and western blot assays.

### Real-time quantitative PCR (qPCR)

The total RNA was extracted from the samples using TRIzol regent (Sigma, USA) according to the manufacturer’s instructions. The RNA concentration was determined by Nanodrop 100 (Thermo Fisher Scientific, USA), and cDNA was synthesized using Hiscript QRT supermix (Vazyme, China). Following this, a 10 μL qPCR reaction was performed following instructions of the SYBR Green mastermixs Kit (Vazyme, China) with the biosystems 7500 sequence detection system. GAPDH was used as the inner control. The relative mRNA levels were calculated using the 2^−ΔΔCt^ method. Primers used for the PCR reaction were listed in Supplementary Table 2.

### Western blotting (WB)

Samples were lysed with ice-cold radioimmunoprecipitation (RIPA) lysis, and their total protein content was quantified using the BCA Protein Assay Kit (HyClone-Pierce, USA). 10% sodium dodecyl sulfate polyacrylamide gel electrophoresis (SDS-PAGE) (Invitrogen, USA) was performed to separate whole-cell lysates. Isolated proteins were transferred to polyvinylidene difluoride (PVDF) membranes. 5% slim milk was incubated with membranes to block nonspecific antigen sites. After that, primary antibodies were incubated with the membranes overnight, followed by the addition of the secondary horseradish peroxidase (HRP)-conjugated antibody, specifically goat anti-rabbit/mouse IgG. Relative protein levels were visualized using enhanced chemiluminescence (ECL, Millipore, USA). The antibodies used in WB were listed in Supplementary Table 3.

### Celigo cell counting assay

Celigo cell counting assays were performed to detect cell viability. Following transfection with the indicated lentivirus, 2 × 10^3^ 786-O or 3 × 10^3^ ACHN cells were seeded into 96-well plates and cultured for 5 consecutive days. The cell number at indicated time points was determined by Celigo image cytometer (Nexcelom Bioscience, USA). The cell proliferative curve was generated based on the obtained cell counts.

### Flow cytometry

Flow cytometry was used to analyze changes in cell apoptosis and cycle distribution. 786-O and ACHN cells were seeded into 6-well plates at a density of 1 × 10^6^ cells per well, followed by transfection with the indicated lentivirus. Cell apoptosis analysis involved harvesting the cells at 90% confluence and resuspending them for Annexin V-APC (eBioscience, USA) staining. Similarly, cells were collected post-transfection and then fixed using 70% ethanol. Subsequently, the cells were stained with a mixed solution of PI and RNaseA (TakaRa, China). Apoptotic cell percentages and distribution in different stages were obtained using flow cytometer (Millipore, USA).

### Wound-healing assay

Wound-healing assays were performed to evaluate cell migration. 786-O and ACHN cells were seeded at a density of 5 × 10^4^ cells per well in 12-well plates and then transfected with indicated lentivirus until reaching 90% confluence. After that, a scratch was generated using a scratch tester, and the cells were maintained in a medium containing 0.5% FBS. The migratory distance at indicated time points was observed by Cellomics (Thermo Fisher Scientific, USA), and the migration rate was calculated based on the measured migratory distance.

### Transwell assay

Cell migration capacity was also evaluated by transwell assays. 786-O and ACHN cells were collected after transfection and resuspended in 100 μL of FBS-free medium in the upper chamber. Meanwhile, the lower chambers were supplemented with 600 μL of 30% FBS, and the upper chamber containing cells were placed onto the lower chamber. Following a 24-hour incubation, non-migrating cells on the lower surface of membrane were gently removed, and the migratory cells were stained with crystal violet for 5 min. The number of migratory cells was determined by a fluorescence microscope.

### Mice xenograft model

Four-weeks-old BALB/c nude mice were purchased from GemPharmatech Co., Ltd. (Jiangsu, China) and randomly divided into two groups: shCtrl group (*n* = 5) and shCALCR group (*n* = 5). ACHN cells transfected with shCtrl or shCALCR were harvested and subcutaneously injected into the flank area of corresponding mice for tumorigenicity. After ten days of inoculation, the widest (W) and the longest (L) diameters of the tumors were measured every 5 days. The tumor volume was calculated using the formula: V = π/6 × L × W^2^. At the end of the experiments, all mice were sacrificed, and tumor tissues were isolated, weighted and photographs of all mice and tumors were taken. CALCR and Ki-67 protein levels were determined by western blot assays and IHC staining as described previously. This animal study was approved by the Ethics Committee of The First Affiliated Hospital of Harbin Medical University.

### Affymetrix human gene chip prime view

The gene expression profile of 786-O cells with or without CALCR depletion was obtained using Affymetrix human Gene Chip Prime View. The differentially expressed genes (DEGs) between shCALCR-depleted and control 786-O cells were screened by criterion of |Fold Change| ≥1.5 and false discovery rate (FDR) <0.05. The DEGs were then visualized using a heat map for Hierarchical Clustering analysis and a volcano plot. Potential downstream targets were analyzed by constructing an interaction network using Ingenuity Pathway Analysis (IPA).

### Bioinformation analysis

The correlation between differentially expressed genes (DEGs) and CALCR expression, as well as the association between DEGs and overall survival in renal clear cell carcinoma patients were determined using renal clear cell carcinoma samples from The Cancer Genome Atlas (TCGA) downloaded from the Genomic Data Commons (GDC) data portal (https://portal.gdc.cancer.gov/). The dataset utilized RNA sequencing data, and the expression values were transformed using the formula log2 (TPM+1). Spearman correlation coefficient was employed to assess the expression correlation, considering a correlation significant if the coefficient was greater than 0 and the *p*-value was less than 0.05. Additionally, prognostic relevance was determined based on a significance threshold of *p* < 0.05.

### Co-immunoprecipitation (Co-IP)

ACHN cell proteins were collected to investigate the interaction between CALCR and CD44. 1.0 mg of total proteins were incubated with anti-CALCR or IgG at 4°C overnight. Subsequently, 20 μL of agarose beads were added and incubated at 4°C for 2 h. After centrifugation, the protein A/G beads were harvested and denatured in IP lysate buffer and 5× loading buffer. Finally, 20 μg protein sample was subjected to WB analysis as described above.

### The ubiquitination assay

The ubiquitination assay was performed as previously described [16]. Briefly, the 786-O and ACHN cells that had been transfected with shCtrl or shCALCR lentivirus were harvested following treatment with 20 μΜ MG132. Cells were then lysed using RIPA buffer and denatured by boiling for 5 min. The process of immunoprecipitation (IP) and western blot analysis was performed in accordance with the aforementioned protocol.

### The human phospho-kinase array analysis

Cell lysates from ACHN cells transfected with shCtrl or shCALCR lentivirus were prepared. Following the manufacturer’s instructions for the human phospho-kinase array kit (Cat. #ARY003C, R&D), the microarray membranes were blocked using array buffer. The cell lysates were then added to the microarray membranes and incubated at 2−8°C for 24 hours. Subsequently, the detection antibody cocktail A (DAC-A) and DAC-B were added to wells containing Part A membranes and Part B membranes and left to incubate for 2 hours. The membranes were then washed and incubated with streptavidin-HRP for 30 min. Finally, the pixel density on the membrane was detected by image lab 6.0 software (Bio-Rad, USA).

### Statistical analysis

Statistical analysis was performed using Graphpad Prism 8.04 and SPSS 22.0 software. Data is shown as the mean ± standard deviations (SD). Statistical analysis between two groups was performed by two-tailed Student’s *t*-test. Statistical analysis of more than two groups were evaluated by one-way analysis of variance (ANOVA) followed by a Tukey post-hoc test. The Sign test and Mann-Whitney U analysis were used for statistical analysis of Tables 1 and 2, respectively. *P*-value of < 0.05 is considered statistically significant. Survival difference in the Kaplan-Meier curves was calculated by a log-rank test.

**Table 1:** Expression of CALCR in renal carcinoma tissues and para-carcinoma tissues was revealed by immunohistochemistry analysis.

**CALCR expression**	**Tumor tissue**		**Para-carcinoma tissue**		***p*-value**
	**Cases**	**Percentage**	**Cases**	**Percentage**	
Low	37	46.8%	61	83.6%	<0.001
High	42	53.2%	12	16.4%	

Abbreviation: CALCR: calcitonin receptor.

**Table 2:** Relationship between CALCR expression and tumor characteristics in patients with renal carcinoma.

**Features**	**No. of patients**	**CALCR expression**		***p*-value**
		**low**	**high**	
All patients	79	37	42	
Age (years)				
≤56	40 (50.63%)	21 (52.50%)	19 (47.50%)	0.31
>56	39 (49.37%)	16 (41.03%)	23 (58.97%)	
Gender				
Male	55 (69.62%)	25 (45.45%)	30 (54.55%)	0.364
Female	24 (30.38%)	12 (50.00%)	12 (50.00%)	
Stage				
I	58 (73.42%)	33 (56.90%)	25 (43.10%)	0.003
II	1 (1.27%)	0 (0)	1 (100%)	
III	4 (5.06%)	1 (25.00%)	3 (75.00%)	
IV	16 (20.25%)	3 (18.75%)	13 (81.25%)	
T Infiltrate				
T1	68 (86.08%)	35 (51.47%)	33 (48.53%)	0.039
T2	4 (5.06%)	1 (25.00%)	3 (75.00%)	
T3	6 (7.59%)	1 (16.67%)	5 (83.33%)	
T4	1 (1.27%)	0 (0)	1 (100%)	
Lymphatic metastasis (*N*)				
N0	74 (93.67%)	37 (50.00%)	37 (50.00%)	0.031
N1	5 (6.33%)	0 (0)	5 (100%)	
Metastasis				
M0	63 (79.75%)	34 (53.97%)	29 (46.03%)	0.012
M1	16 (20.25%)	3 (18.75%)	13 (81.25%)	

### Availability of data and materials

The data generated in this study are available within the article and its supplementary data files.

## RESULTS

### Overexpression of CALCR predicts poor prognosis for patients with renal carcinoma

**Figure 1:**
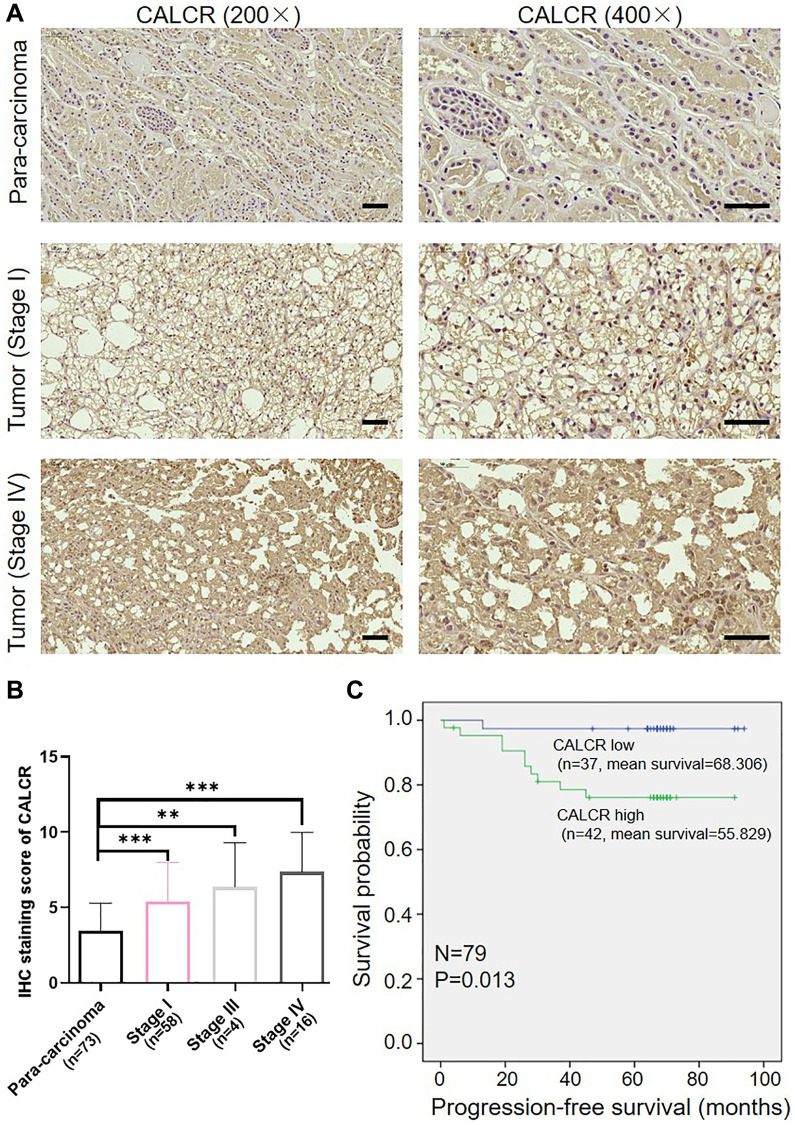
**Overexpression of CALCR predicts poor prognosis for patients with renal carcinoma.** (**A**) Representative IHC images of CALCR expression in human RCC tissues and normal para-carcinoma tissues. Scale bar represent 50 μm. (**B**) Quantification of CALCR expression in IHC staining. (**C**) Kaplan-Meier analysis indicated that high CALCR expression was correlated with short PFS. Results were presented as mean ± SD. ^**^p < 0.01, ^***^p < 0.001. Abbreviations: CALCR: calcitonin receptor; IHC: immunohistochemical; PFS: progression-free survival.

We first determined the expression of CALCR in RCC using a human tissue chip containing 79 of RCC tissues and 73 of normal para-carcinoma tissues by IHC analysis. As the IHC figures shown, CALCR was markedly upregulated in RCC tissues, especially in advanced RCC tissues, compared with normal para-carcinoma tissues (para-carcinoma vs. stage I, *P* < 0.001; para-carcinoma vs. stage III, *P* = 0.004; para-carcinoma vs. stage IV, *P* < 0.001) (Figure 1A, 1B, Table 1). Besides, correlation analysis between CALCR expression and tumor characteristics in RCC patients suggested that the overexpression of CALCR was positively associated with tumor stage (*P* = 0.003), T infiltration (*P* = 0.039), metastasis (*P* = 0.012) and lymphatic metastasis (*P* = 0.031) (Table 2), which was confirmed by Spearman correlation analysis (Table 3). In addition, Kaplan-Meier survival analysis showed that RCC patients with higher expression level of CALCR had a shorter progression-free survival (PFS) (*P* = 0.013) (Figure 1C), implying the predictive value of CALCR for poor prognosis in patients with RCC. These results indicated that CALCR exerted a promoting role in renal carcinoma progression.

**Table 3:** Spearman correlation analysis between CALCR expression and tumor characteristics in patients with renal carcinoma.

**Tumor characteristics**	**Index**	**CALCR**
T infiltrate	Spearman correlation	0.234
	Significance (two-tailed)	0.038
	*N*	79
Stage	Spearman correlation	0.332
	Significance (two-tailed)	0.003
	*N*	79
Metastasis	Spearman correlation	0.284
	Significance (two-tailed)	0.011
	*N*	79
Lymphatic metastasis (*N*)	Spearman correlation	0.244
	Significance (two-tailed)	0.030
	*N*	79

### CALCR promotes malignant phenotypes of renal carcinoma cells

**Figure 2:**
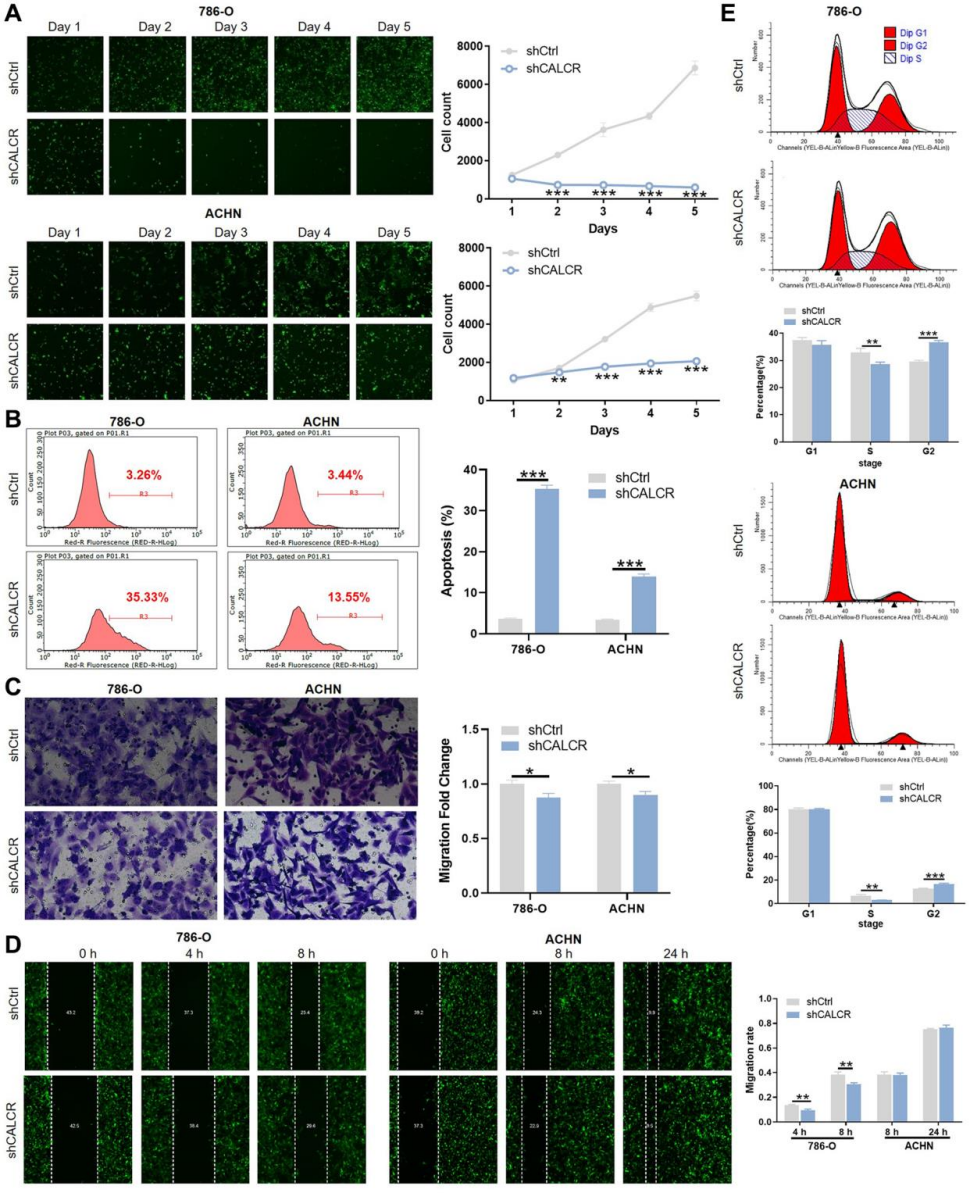
**CALCR promotes malignant phenotypes of renal carcinoma cells.** (**A**) Cell proliferative capacities of 786-O and ACHN cells transfected with shCtrl or shCALCR lentivirus were evaluated by Celigo cell count assay. (**B**) Alterations of cell apoptosis in 786-O and ACHN cells transfected with shCtrl or shCALCR lentivirus were analyzed by flow cytometry. (**C**, **D**) Cell migratory abilities of 786-O and ACHN cells transfected with shCtrl or shCALCR lentivirus were assessed by (**C**) transwell assay and (**D**) wound-healing assay. (**E**) The effect of CALCR on cell cycle distribution was analyzed by flow cytometry. The representative images were selected from at least 3 independent experiments. Results were presented as mean ± SD. ^*^p < 0.05, ^**^p < 0.01, ^***^p < 0.001.

To confirm the biological significance of CALCR in RCC development, we silenced CALCR in 786-O and ACHN RCC cell lines using CALCR shRNAs. According to the results of qPCR assays (Supplementary Figure 1A), shCALCR-2 was identified as the most effective CALCR knockdown shRNA and, accordingly, lentiviruses containing shCALCR-2 sequences were employed consistently throughout the study. As a negative control, a lentiviral vector carrying scramble RNA (shCtrl) was utilized. Further, compared with shCtrl group, the expression levels of CALCR mRNA (*P* < 0.001, 786-O; *P* = 0.016, ACHN) and protein in 786-O cells and ACHN cells transfected with shCALCR-2 were down-regulated, indicating that the CALCR knockdown cell model was successfully constructed (Supplementary Figure 1B, 1C). The influence of CALCR on malignant phenotypes of RCC cells, including cell proliferation, anti-apoptosis, migration, were then evaluated. The results of Celigo cell count assay revealed that CALCR knockdown significantly impaired proliferative ability of 786-O and ACHN cells compared to the negative control group (*P* < 0.001) (Figure 2A). In contrast, CALCR depletion remarkably promoted 786-O and ACHN cells apoptosis, indicating the inhibition role of CALCR on cell apoptosis (*P* < 0.001) (Figure 2B). Cell migratory alterations were observed by transwell and wound healing assays. As shown in Figure 2C, the transwell assays suggested that migratory capacity of renal cancer cells was apparently attenuated upon CALCR knockdown (*P* = 0.015, 786-O; *P* = 0.012, ACHN). The wound healing results of 786-O cells were consistent with those of transwell assays (*P* =0.004, 4 h; *P* = 0.007, 8 h) (Figure 2D). In addition, we observed that CALCR depletion arrested 786-O and ACHN cell cycle in G2 phase (*P* < 0.001, 786-O; *P* < 0.001, ACHN), whereas significantly decreased the proportion of S-stage cells (*P* = 0.009, 786-O; *P* = 0.002, ACHN) (Figure 2E). Collectively, these results suggested that CALCR knockdown could inhibit the oncogenic features of malignant proliferation and migration in RCC cells, and induce cell apoptosis and cell cycle arrest.

### CALCR is indispensable for *in vivo* tumor formation

**Figure 3:**
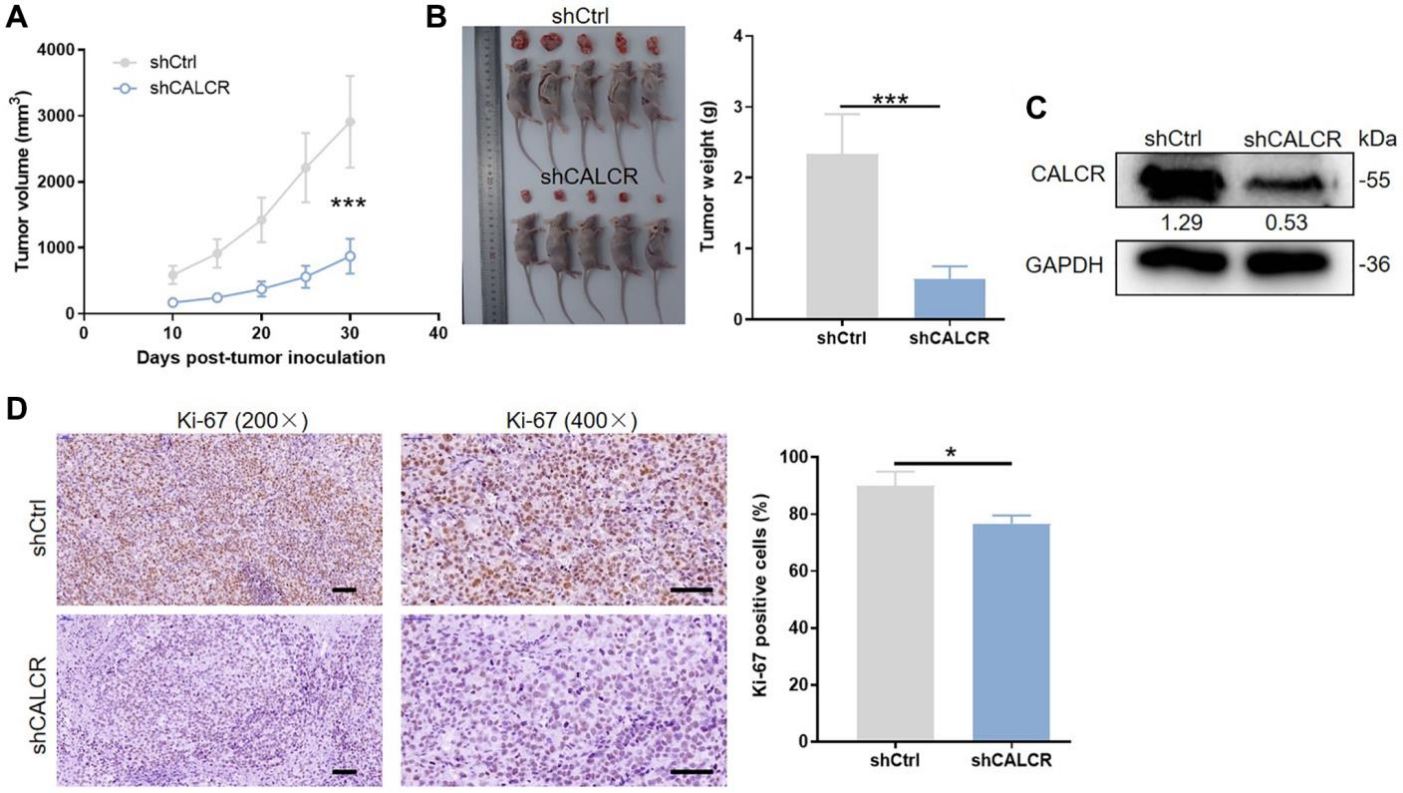
**CALCR is indispensable for *in vivo* tumor formation.** (**A**) Tumor size was measured as shown, and the tumor growth curve was graphed based on tumor size. (**B**) All mice and corresponding tumor tissues in shCtrl group and shCALCR group was shown as the figure. Right panel presented the tumor. (**C**) CALCR protein level in tumor tissues isolated from mice in shCtrl group and shCALCR group was determined by western blot assay. (**D**) Representative Ki-67 staining images of tumor tissues in shCtrl group and shCALCR group. The quantitative statistical analysis for Ki67 expression was shown on the right. Scale bar represent 50 μm. Results were presented as mean ± SD. ^***^p < 0.001.

To identify whether CALCR is essential for *in vivo* tumor formation, the ACHN cells with CALCR stable knockdown were subcutaneously injected into flank area of nude mice. 10 days after cell inoculation, tumor volume was obtained for generating a tumor growth curve. As shown in Figure 3A, tumors from mice in shCALCR group were slower growing than that in shCtrl group (*P* < 0.001). Consistently, the tumor weight of shCALCR group mice was apparently lighter compared to the tumor weight of shCtrl group mice (*P* < 0.001) (Figure 3B). The western blot assays of tumor tissues showed that CALCR was effectively knocked down relative to the corresponding control group (Figure 3C). By performing Ki-67 staining of tumor tissues, we found that the downregulation of CALCR accompanied a reduction in expression of Ki-67 (*P* = 0.016), implying the inhibition of CALCR on tumor cell proliferation (Figure 3D). Taken together, we demonstrated that CALCR was critical for *in vivo* tumor growth of RCC cells.

### CALCR stabilizes CD44 via inhibiting its ubiquitination

**Figure 4:**
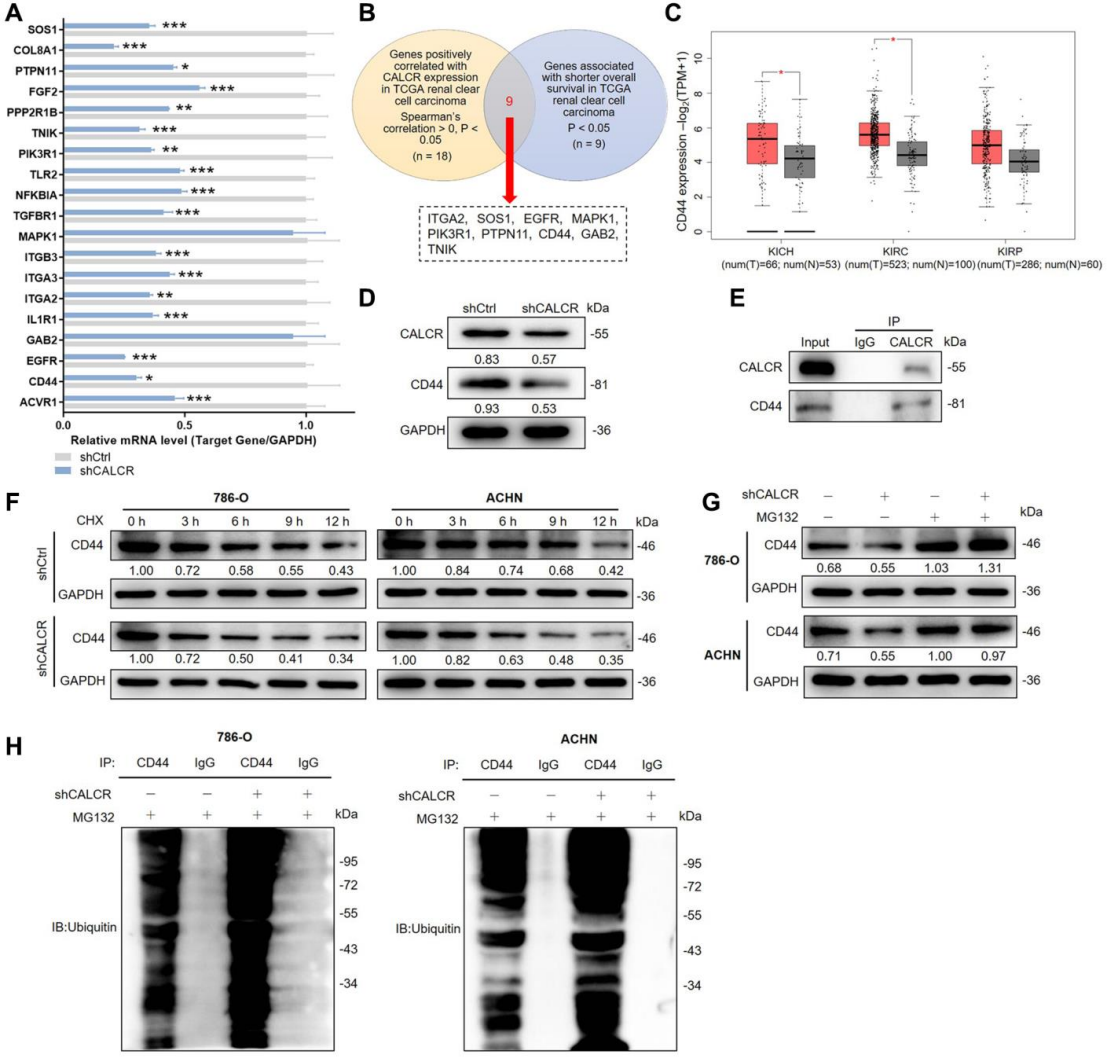
**CALCR stabilizes CD44 via inhibiting its ubiquitination.** (**A**) Relative mRNA levels of several potential target genes were detected by qPCR analysis in ACHN cell with CALCR depletion. (**B**) Venn diagram showing the DEGs correlated with CALCR expression and shorted OS in TCGA renal clear cell carcinoma. (**C**) Relative mRNA expression of CD44 from TCGA renal cancer (KICH, KIRC and KIRP). (**D**) Relative protein levels of CALCR and CD44 in 786-O cells with CALCR deficiency were determined by western blot assays. (**E**) Co-IP analysis suggested the endogenous binding between CALCR and CD44. (**F**) Relative CD44 protein level in CALCR-depleted 786-O and ACHN cells treated with CHX was analyzed by western blot assays. (**G**) Relative CD44 protein level in CALCR-depleted 786-O and ACHN cells treated with MG132 was determined by western blot assays. (**H**) The ubiquitin of CD44 immunoprecipitated by anti-CD44 or IgG antibody was detected. GAPDH served as an internal control in qPCR and western blot assays. Results were presented as mean ± SD. ^*^p < 0.05, ^**^p < 0.01, ^***^p < 0.001. Abbreviations: DEGs: differentially expressed genes; OS: overall survival; TCGA: the Cancer Genome Atlas; CD44: cluster of differentiation; KICH: kidney chromophobe; KIRC: kidney renal clear cell carcinoma; KIRP: kidney renal papillary cell carcinoma; CHX: Cycloheximide.

To gain an in-depth mechanistic view of CALCR-induced RCC progression, we further examined potential proteins that could interact with CALCR. The results of human gene expression profiling microarray sequencing suggested that a total of 403 genes were upregulated and 1011 genes were downregulated along with CALCR depletion (Supplementary Figure 1D, 1E). By qPCR analysis, we validated the differential expression of top-ranked 19 DEGs (Figure 4A). Furthermore, we conducted correlation analyses between these 19 DEGs and CALCR, as well as prognostic assessments correlating with these 19 DEGs with overall survival, utilizing The Cancer Genome Atlas (TCGA) renal clear cell carcinoma data (https://portal.gdc.cancer.gov/). The results showed that 9 DEGs (ITGA2, SOS1, EGFR, MAPK1, PIK3R1, PTPN11, CD44, GAB2, TNIK) were simultaneously positively correlated with CALCR expression, and were associated with shorter overall survival in renal clear cell carcinoma patients (Figure 4B). Subsequently, we assessed the differential expression of these 9 genes in KICH, KIRC, and KIRP from TCGA cohort through GEPIA (http://gepia2.cancer-pku.cn). Notably, CD44 emerged as the only gene consistently showing elevated expression across all three renal cancer types, with statistically significant differences in TCGA-KICH and TCGA-KIRC (Figure 4C). CD44 has been implicated in various cancers, including its role as a potential prognostic marker in RCC [17, 18]. Therefore, we selected CD44 as a key target for CALCR in renal cancer progression. Furthermore, the western blotting was used to investigate CD44 expression after CALCR knockdown in renal cancer cell, and we found that CD44 protein expression was inhibited upon CALCR depletion (Figure 4D). These findings collectively emphasize CD44 as a critical target through which CALCR promotes renal cancer progression.

Ubiquitination is a post-translational modification of proteins and plays a crucial regulatory role in cell survival, differentiation, and cycle distribution [19]. Herein, we observed that CALCR could bind CD44 by Co-IP analysis (Figure 4E), and CALCR suppression in 786-O and ACHN led to a decrease of CD44 protein stability by shortening CD44 degradation half-life under Cycloheximide (CHX) treatment, implying that CALCR could regulate CD44 expression level via its protein degradation (Figure 4F). To determine whether CALCR-mediated protein degradation of CD44 was completed by the ubiquitin-proteasome pathway, we treated 786-O and ACHN cells transfected with indicated lentivirus with a proteasome inhibitor MG132. The results of western blot assays suggested that MG132 apparently restored CD44 protein levels in CALCR-depleted 786-O and ACHN cell lines, indicating that the ubiquitin-proteasome pathway was involved in CALCR-induced CD44 degradation (Figure 4G). Subsequently, the ubiquitination assay was performed to validate this finding. The data showed that CALCR silencing in 786-O cell significantly increased CD44 ubiquitination, and the same result was obtained from the ACHN cell line (Figure 4H). Collectively, we suggested that CALCR could stabilize CD44 by inhibiting its ubiquitination in RCC cells.

### CALCR mediated renal carcinoma progression is depended on CD44

**Figure 5:**
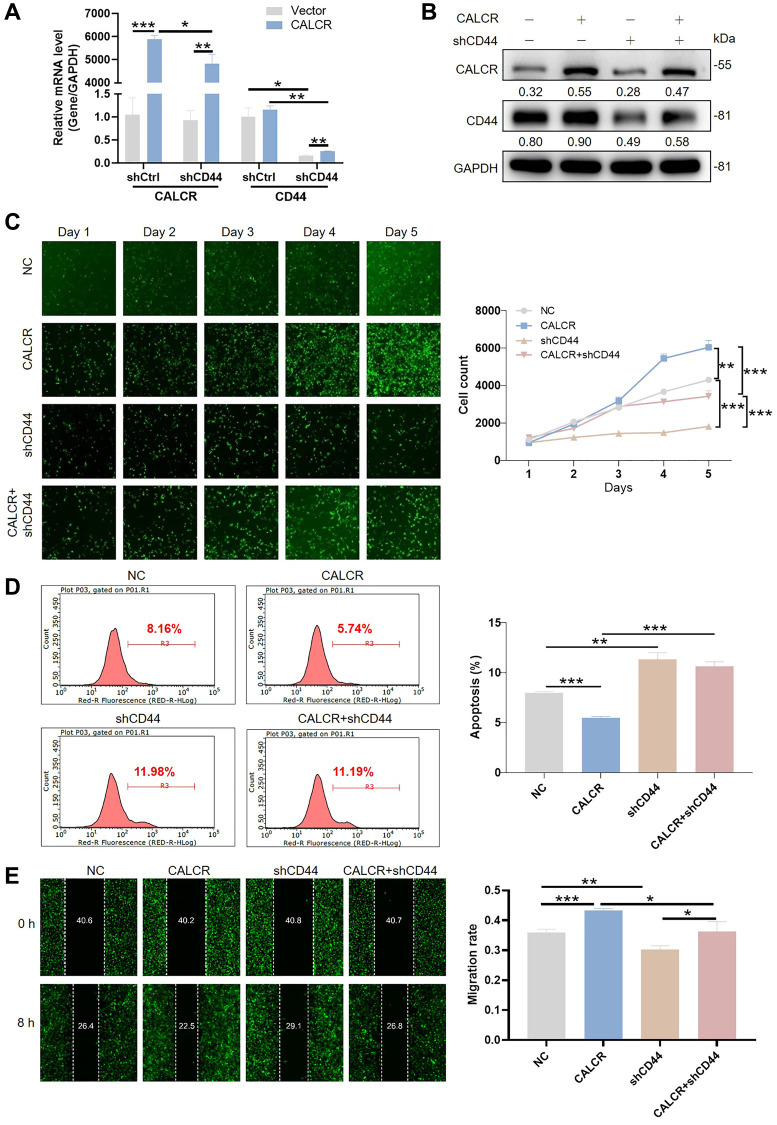
**CALCR mediated renal carcinoma progression is depended on CD44.** CALCR overexpression and CD44 depletion in 786-O cell were confirmed by (**A**) qPCR and (**B**) western blot assays. GAPDH served as an internal control in qPCR and western blot assays. (**C**) Cell viability in 786-O cell transfected with indicated lentiviruses was assessed by Celigo cell count assay. (**D**) Cellular apoptosis in 786-O cell transfected with indicated lentiviruses was evaluated using flow cytometry. (**E**) Cell migration was analyzed by wound-healing assays. The representative images were selected from at least 3 independent experiments. Results were presented as mean ± SD. ^*^p < 0.05, ^**^p < 0.01, ^***^p < 0.001.

Given the regulatory effect of CALCR on CD44 expression, we further explored whether the tumor promotion of CALCR required CD44 expression. We generated the shRNAs targeting CD44 and screened shCD44-1 with the best knockdown efficiency to downregulate CD44 expression in CALCR-overexpressed 786-O cells (Supplementary Figure 1F), and detected the promoting role of CALCR on RCC cells. The mRNA and protein expression level of CALCR and CD44 was confirmed in cells transfected with CALCR and shCD44 (Figure 5A, 5B). The Celigo cell count assay showed that CD44 knockdown significantly impaired CALCR-induced cell proliferation (CALCR + shCD44 group vs. CALCR group, *P* < 0.001) (Figure 5C). Conversely, the inhibition of CALCR on renal cancer cell apoptosis was remarkably attenuated by depleting CD44 expression (CALCR + shCD44 group vs. CALCR group, *P* < 0.001) (Figure 5D). In addition, the wound healing assay revealed that CD44 deficiency decreased the cell migration ability promoted by CALCR (CALCR + shCD44 group vs. CALCR group, *P* = 0.027) (Figure 5E). These rescue experiments suggested CD44 was indispensable for CALCR-induced RCC development.

### The JNK and STAT1 signaling pathway might contribute to renal carcinoma progression

**Figure 6:**
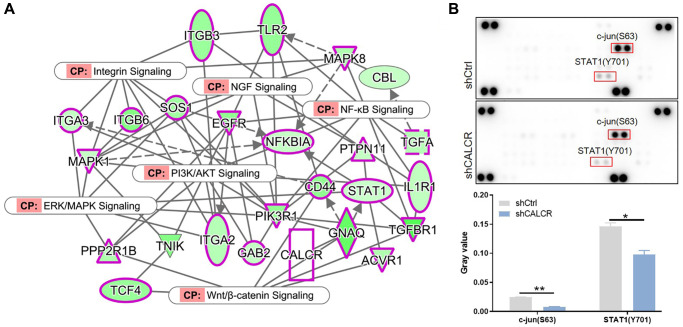
**Possible signaling pathways that contribute to renal carcinoma progression.** (**A**) IPA analysis identified the interaction between CALCR and NF-κB signaling, integrin signaling, PI3K/AKT signaling, ERK/MAPK signaling, Wnt/β-catenin signaling and NGF signaling. (**B**) Human phospho-kinase array analysis suggested the significant changes in phosphorylation levels of c-jun and STAT1 after silencing CALCR. Results were presented as mean ± SD. ^*^p < 0.05, ^**^p < 0.01. Abbreviations: IPA: ingenuity pathway analysis; NF-κB: nuclear factor-kappa B; PI3K: phosphoinositide 3-kinase; AKT: protein kinase B; ERK: extracellular signal-regulated kinase; MAPK: mitogen-activated protein kinase; Wnt: wingless-related integration site; NGF: nerve growth factor; STAT1: signal transducer and activator of transcription 1.

To gain more insight of mechanism by which CALCR exacerbates renal cancer progression, the possible signaling pathways involved in this process was explored. We performed the interaction network analysis based on ingenuity pathway analysis (IPA) software, the results suggested that CALCR could indirectly affect NF-κB signaling, integrin signaling, PI3K/AKT signaling, ERK/MAPK signaling, Wnt/β-catenin signaling and NGF signaling through other intermediate molecules (Figure 6A). Moreover, we further analyzed the phosphorylation levels of 37 kinases using a human phospho-kinase array. We found that the phosphorylation of c-jun (*P* = 0.002) and STAT1 (*P* = 0.032) was significantly inhibited in CALCR-depleted cells compared to the respective control cells (Figure 6B), reminding that c-jun and STAT1 related signaling pathways may be involved in CALCR induced renal carcinoma development. The c-Jun N-terminal Kinases (JNKs) are members of the mitogen-activated protein kinase (MAPK) family and have been identified as a key oncogenic signaling nodes in several cancers [15]. STAT1 is a transcription factor belonging to the signal transducer and activator of transcription (STAT) family. In response to cytokines, STAT1 functions as a tumor suppressor or tumor promoter in various cancer types [20]. Therefore, we inferred that the JNK and STAT1 signaling were possible signaling pathways that contributed to CALCR-mediated RCC progression.

## DISCUSSION

RCC is a highly aggressive malignancy in the field of urology, exhibiting an increasing incidence rate and a high rate of metastasis [16]. Despite advancements in imaging techniques, nearly 30% of patients are already diagnosed with distant metastases at the time of initial diagnosis [1]. Moreover, the 5-year survival rate for patients with metastatic RCC is a mere 10% [19]. One of the main challenges in current therapies is the lack of effective biomarkers that can accurately predict RCC progression [21]. Therefore, it is crucial to unravel the mechanisms responsible for RCC progression in order to develop novel therapeutic strategies that can enhance the survival rate of RCC patients. Herein, we have identified the overexpression of CALCR in human RCC tissues. Furthermore, the upregulation of CALCR has been found to be indicative of a poor prognosis for patients with RCC. CALCR holds potential as a novel therapeutic target in RCC.

CALCR is a G protein-coupled receptor known to play a critical role in calcium homeostasis [8, 14]. In addition to its involvement in calcium regulation, there is evidence suggesting that CALCR acts as both tumor suppressor and an oncogene, influencing cell survival, apoptosis and cell cycle progression [22]. For example, Nakamura M. et al. reported that CALCR significantly inhibited *in vivo* tumor growth of MDA-MB-231 breast cancer cells with constitutively phosphorylated ERK1/2 expression [23]. Conversely, in PC3 prostate cancer cells, CALCR promoted proliferation and invasion while suppressing apoptosis through its interaction with zonula occludens-1 and inducing PKA-mediated disassembly of tight junctions [24, 25]. It should be noted that Calcitonin and CALCR co-expression was observed in PC3M cells, which enhanced the invasiveness in this cancer context [26, 27]. In our study, we investigated the role of CALCR in RCC cells and found that knocking down CALCR inhibits several oncogenic features, including cell proliferation, migration and anti-apoptosis properties. Additionally, our results suggested that CALCR affects RCC cell cycle distribution, specifically reducing the proportion of cells in the S stage and arresting them in the G2 stage. Moreover, CALCR depletion restricted *in vivo* tumor growth of ACHN cells. Our findings were consistent with the study of CALCR in the majority of human cancers. Therefore, this research supports the notion that CALCR served as an oncogene in RCC. While these results highlight the promoting role of CALCR in RCC, the precise underlying mechanism through which CALCR contributes to RCC progression remains unclear.

To further elucidate the in-depth mechanisms underlying CALCR-mediated RCC development, we investigated potential proteins interactions with CALCR in RCC cells. Our screening data revealed that CD44 was among the probable downstream molecules, and subsequent Co-IP experiments confirmed the binding between CD44 and CALCR. CD44 is a transmembrane glycoprotein primarily expressed on non-kinase cell surface [28]. It is located on human chromosome 11 or murine chromosome 2 and is known by various aliases, including Pgp-1/Ly-24, In (Lu)-related protein p80, HUTCH-1, ECMRIII, Hermes antigen, and hyaluronate receptor [29, 30]. CD44 has been extensively identified as a cancer stem cell marker in multiple cancer types [31]. Elevated expression of CD44 has been observed in various cancers such as prostate cancer, ovarian cancer, gallbladder cancer, oral squamous cell carcinoma and gastric cancer, demonstrating correlation with malignant biological phenotypes and an unfavorable prognosis [32, 33].

Ubiquitination is an imperative post-translation processes in eukaryotes, and we found that CALCR enhances CD44 protein stability via prolonging its degradation half-life, suggesting that CALCR might regulate CD44 protein level through the ubiquitin-proteasome protein degradation pathway. Therefore, we explored the functional significance of CD44 in CALCR-induced RCC progression. In clear cell RCC, CD44 confers a significant advantage in invasiveness and metastatic to cells, and it also serves as a predictor of a poor prognosis [34–36]. In line with these observations, our rescue assays demonstrated that CD44 depletion markedly hindered the promoting role of CALCR in RCC cell growth, migration and anti-apoptosis, emphasizing the essential involvement of CD44 in CALCR-driven RCC progression.

Our signaling screening data indicated that the JNK and STAT1 signaling pathways could potentially contribute to CALCR-mediated RCC progression. JNK signaling has been extensively studied in various tumor types, and its abnormal activation has been implicated in the development of tumor [37, 38]. On the other hand, STAT1 is generally known to act as a suppressor in various malignancies [20, 39]. Interestingly, in RCC, the activation of STAT1 has been found to frequently decrease radiosensitivity [40–42]. The specific connection between CALCR and JNK/STAT1 signaling is largely unclear. However, there is evidence that calcitonin can increase CD44 variant RNA and protein levels via p38 pathway in CALCR-positive prostate cancer cell [43]. This report supports the hypothesis that the JNK signaling may potentially serve as one of the downstream pathways in CALCR-mediated RCC development.

In conclusion, our study has uncovered a novel biological connection between CALCR and CD44 expression in RCC. Analysis of human samples revealed the overexpression of CALCR in RCC, and this overexpression was positively associated with the malignant progression of RCC. Through our biological studies, we also observed that knockdown of CALCR inhibited RCC cell proliferation, migration and induced apoptosis and cycle arrest *in vitro*. Furthermore, the suppression of tumor growth induced by CALCR depletion was also observed in animal models. Notably, our findings indicated that CALCR plays a role in the deubiquitination of CD44 protein, leading to enhanced CD44 expression in RCC. Considering these significant findings, targeting CALCR may be a promising therapeutic strategy for treating RCC patients. However, specific mechanisms and possible pathways for the regulation of CD44 ubiquitination by CALCR require more studies to illustrate.

## Supplementary Materials

Supplementary Figure 1

Supplementary Tables
