# Supplementary material for: CALCR exacerbates renal cell carcinoma progression via stabilizing CD44
Source: Aging (Albany NY). 2024 Jul 9;16(13):10765–83. doi: 10.18632/aging.205586 (PMC11272109; doi:10.18632/aging.205586)
Supplement: Supplementary Figure 1 [file aging-16-205586-s001.pdf]

SUPPLEMENTARY FIGURE

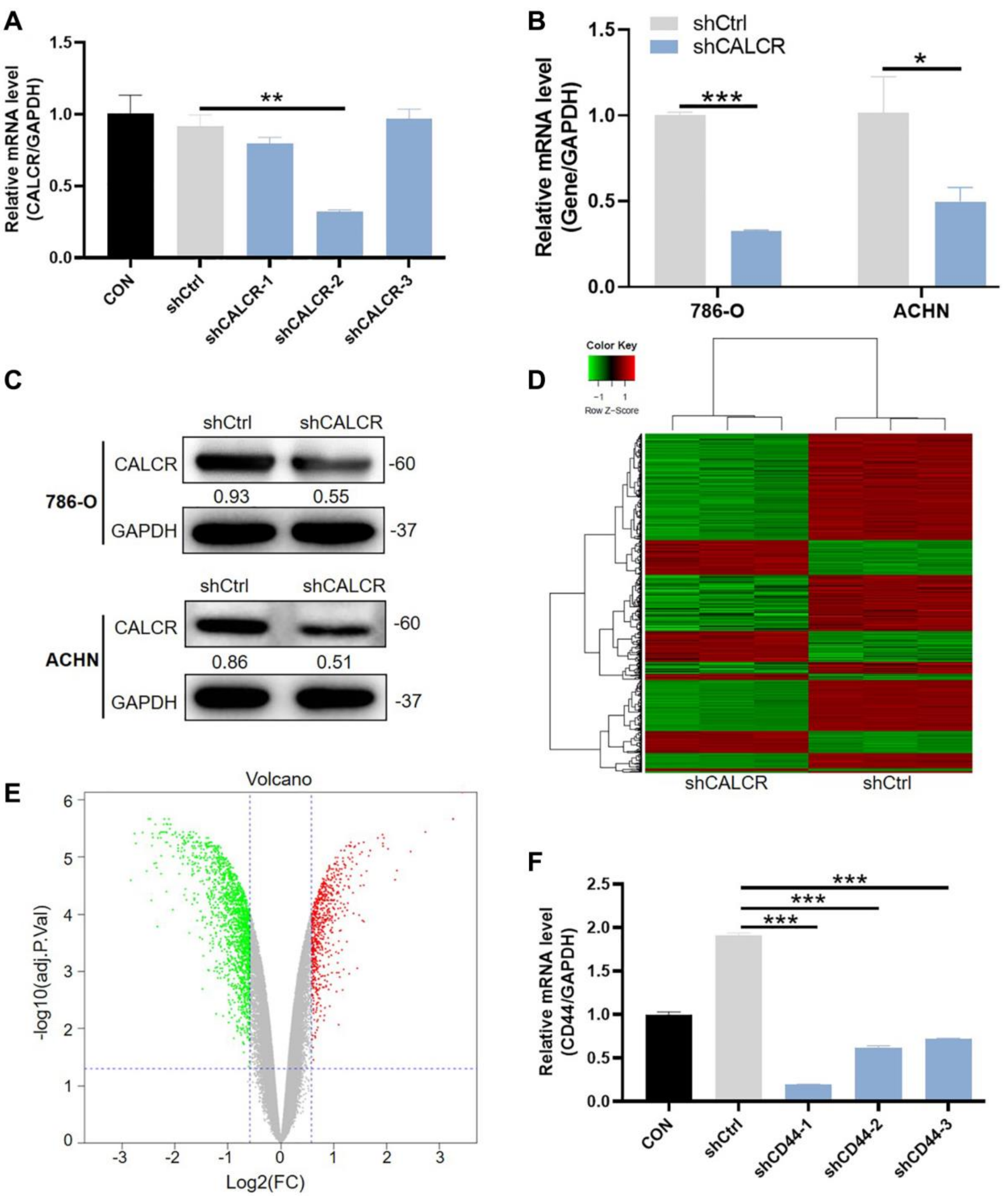

Supplementary Figure 1. (**A**) Screening for the shCALCR with the best knockdown efficiency (shCALCR-2). (**B**) CALCR deficiency in 786-O and ACHN cells were confirmed by qPCR and (**C**) western blot assays. (**D**) The heat map and (**E**) volcano plot showed the DEGs between shCALCR-depleted and control 786-O cells. Red represents the upregulated genes, green represents the downregulated genes. (**F**) Screening for the shCD44 with the best knockdown efficiency (shCD44-1).
