# Supplementary material for: CALCR exacerbates renal cell carcinoma progression via stabilizing CD44
Source: Aging (Albany NY). 2024 Jul 9;16(13):10765–83. doi: 10.18632/aging.205586 (PMC11272109; doi:10.18632/aging.205586)
Supplement: Supplementary Tables [file aging-16-205586-s002.pdf]

SUPPLEMENTARY TABLES

Supplementary Table 1. Target sequences of shRNAs.

Gene	No.	Target sequence (5′–3′)
CALCR	shCALCR-1	GAGGAATGAACCAGCCAACAA
CALCR	shCALCR-2	TGGGTCATTCTTTGTCAATTT
CALCR	shCALCR-3	CAAATCAAACCTATCCAACAA
CD44	shCD44-1	AAGCTCTGAGCATCGGATTTG
CD44	shCD44-2	TTGAATATAACCTGCCGCTTT
CD44	shCD44-3	CCGCTGACCTCTGCAAGGCTT

Abbreviations: CALCR: calcitonin receptor; CD44: cluster of differentiation.

Supplementary Table 2. Primers used in qPCR.

Primer name	Forward primer sequence (5′–3′)	Reverse primer sequence (5′–3′)
CALCR	CCGGTGAGCTGCAAGATTT	AGCCACGACAATGAGTGTATGA
ACVR1	TCTGTAGTGTTCGCAGTATGT	ATTGAGGCGTTCTTGGTT
CD44	TGGGTTCATAGAAGGGCACG	ATACTGGGAGGTGTTGGATGTG
EGFR	ATGAGGACATAACCAGCCACC	AGGCACGAGTAACAAGCTCAC
GAB2	TGGACAACAGCCGACTTCA	GGAAAGAGCCAACTCCATCAC
IL1R1	GTGGCTGAAAAGCATAGAGGG	GTCTCATTAGCTGGGCTCACA
ITGA2	GGCGACGAAGTGCTACGAAA	CCCAAGAACTGCTATGCCAAAC
ITGA3	CCCACTCACTGCCCACAAGG	CCACAGTCACTCCAAGCCACAT
ITGB3	CGGCAGGTGGAGGATTAC	CTTTCGCATCTGGGTGGC
MAPK1	GACTGGACGTGCTCAGACAT	CCTCCAAACGGCTCAAAGGA
TGFBR1	GTCATCACCTGGCCTTGGTC	GGTCCTCTTCATTTGGCACTC
NFKBIA	CTCCATCCTGAAGGCTACCAA	GCACCCAAGGACACCAAAAG
TLR2	TGACTCTACCAGATGCCTCCCT	TTGCCACCAGCTTCCAAAG
PIK3R1	GAAGAAATTGGCTGGTTAAATGG	CTGGTGCAACAGGAAGAGGC
TNIK	CGACATACCCAGACTGATAC	AATACTGCCGCTGAAACT
PPP2R1B	TTAGCAAGTGGGGATTGGTTC	GCATTTGATGCCCTGGGATAG
FGF2	AGCGACCCTCACATCAAGCT	GCCAGGTAACGGTTAGCACA
PTPN11	CGGCAAGTCTAAAGTGACCC	AATCAAACCGTTCTCCTCCAC
COL8A1	ATTCCTCCTCAGATGCCACCA	GGACCTTGTTCCCCTCGTAAA
SOS1	GCCAGCCTCATTGTCCCTAA	ACTGAAGGGGGTCCAATGTG
GAPDH	TGACTTCAACAGCGACACCCA	CACCCTGTTGCTGTAGCCAAA

Abbreviations: ACVR1: activin a receptor type 1; CD44: cluster of differentiation 44; EGFR: epidermal growth aactor receptor; GAB2: GABA receptor subunit beta; IL1R1: interleukin 1 receptor type 1; ITGA2: integrin subunit alpha 2; ITGA3: integrin subunit alpha 3; ITGB3: integrin subunit beta 3; MAPK1: mitogen-activated protein kinase 1; TGFBR1: transforming growth factor beta receptor type 1; NFKBIA: nuclear factor of kappa light polypeptide gene enhancer in B-Cells inhibitor alpha; TLR2: toll-like receptor 2; PIK3R1: phosphoinositide-3-kinase regulatory subunit 1; TNIK: TRAF2 and NCK interacting kinase; PPP2R1B: protein phosphatase 2 regulatory subunit alpha B; FGF2: fibroblast growth factor 2; PTPN11: protein tyrosine phosphatase non-receptor type 11; COL8A1: collagen type VIII alpha 1 chain; SOS1: son of sevenless homolog 1.

Supplementary Table 3. Antibodies applied in IHC, WB, and ubiquitination assays.

Antibody	Diluted multiples	Company	Catalog No.	Use
CALCR	1:50	Abcam	ab230500	IHC
CALCR	1:1000	Abcam	ab230500	WB
CD44	1:2000	Abcam	ab157107	WB
CD44	1:2000	Proteintech	15675-1-AP	Co-IP
GAPDH	1:3000	Proteintech	60004-1-lg	WB
Goat anti-Rabbit	1:3000	Beyotime	A0208	WB
Goat anti-Mouse	1:3000	Beyotime	A0216	WB
Ubiquitin	1:2000	CST	3936S	Ubiquitination
